# Predictive factors of postoperative complications related to free flap reconstruction in head and neck cancer patients admitted to intensive care unit: a retrospective cohort study

**DOI:** 10.1186/s12871-024-02649-9

**Published:** 2024-07-29

**Authors:** Shujing Yu, Kaiyuan Wei, Dawei Zhou, Qing Lin, Tong Li

**Affiliations:** 1 Department of Critical Care Medicine, Beijing Tongren Hospital, Capital Medical University, No. 2, Xihuan South Road, Daxing District, Beijing, China; 2 School of Basic Medicine, Capital Medical University, Beijing, China

**Keywords:** Head and neck cancer surgery, ICU, Free flap reconstruction, Operative complication, Fluid input

## Abstract

**Background:**

The epidemiology and risk factors for postoperative complications related to free flap reconstruction in head and neck cancer patients admitted to the Intensive Care Unit (ICU) are unknown.

**Methods:**

We performed a retrospective cohort study of patients with free flap reconstruction of head and neck cancer between September 2015 and April 2023 admitted to the ICU of Beijing Tongren Hospital. The univariate and multivariate analyses were used to explore the risk factors for postoperative complications related to free flap reconstruction admitted to ICU, including flap necrosis, bleeding, fistula, and infection.

**Results:**

A total of 239 patients were included in this study, and 38 (15.9%) patients had postoperative complications related to free flap reconstruction. The median length of ICU stay was 1 day (interquartile range, 1–2 days). Multivariate analysis found that low BMI (*P* < 0.001), high postoperative CRP (*P* = 0.005), low hemoglobin (*P* = 0.012), and inadequate fluid intake (*P* < 0.05) were independent risk factors for complications.

**Conclusions:**

Postoperative complications related to free flap reconstruction were common in this ICU population. Careful fluid management and monitoring of CRP and hemoglobin levels may reduce complications.

## Introduction

Head and neck cancer is the seventh most common cancer, accounting for 3% of all cancers [1]. To completely remove middle-and-late-stage head and neck cancer, radical resection combined with skin flap transplantation is often used [1]. Free flap reconstruction provides vascularized tissue transferred from a distant donor site on a patient’s body to a recipient site, markedly improving wound closure and protecting head and neck structures [2]. Over the past decade, technological advances have led to the development of new devices for more precise surgery. These devices are based on improved maneuverability, minimally invasive approaches, and magnification of the surgical field [3]. Such as the 3D exoscope has been considered to perform reconstructive head and neck free flap techniques, mostly when performing microvascular anastomoses [3]. Even though free flaps have been widely used in head and neck surgery, postoperative complications related to flap reconstruction such as flap necrosis, hemorrhage, pharyngeal fistula, and so on could be as high as 31% [4]. Several literatures [5–7] discussed the analysis of factors associated with postoperative complications of free flaps in patients with head and neck cancer. However, the risk factors have not been fully elucidated, especially for patients with high surgical risk after surgery who are required to be admitted into the intensive care unit (ICU) for close nursing and monitoring.

Fluid balance is paramount important for ICU patients. Due to the lack of lymphatic drainage and ischemia, free flaps are prone to fluid overload, especially during periods of vascular clamping. Additionally, insufficient infusion can exacerbate ischemia-reperfusion injury, accelerate the inflammatory response, and even lead to flap failure [8]. There have been several research studies in this area, but the conclusions lack consistency. Farwell et al. [9]. and Patel et al. [10]. found no significant correlation between intraoperative fluid administration and the incidence of medical complications. Haughey BH’s study [11] revealed that significant crystalloid administration during surgery was identified as a risk factor for major medical complications, whereas Zhong et al.[12] came to the opposite conclusion. Therefore, further research is necessary to elucidate the optimal fluid management strategy.

Venous thromboembolism (VTE) is a common cardiovascular disease that can result in significant morbidity, including painful leg swelling, chest pain, shortness of breath, and even death [13]. VTE includes deep vein thrombosis (DVT) and pulmonary embolism (PE), both of which are common complications in surgical patients [14]. Patients who undergo free flaps transfer are at high risk for VTE [15]. Thai reported the incidence of VTE in patients with head and neck cancer after resection and microvascular reconstruction ranged from 1.4–5.8% [16]. Vinita Bahl et al. [17]. showed a higher incidence of VTE in patients with free flaps who did not receive chemoprophylaxis was 7.7%. In contrast, based on previous studies, the reported overall incidence of postoperative complications after flap surgery ranges from 11–59% [18, 19]. Calf muscle veins are distal lower extremity veins that are nonpaired and not associated with named tibial arteries [20]. Calf muscle venous thrombosis (CMVT) is the most common form of distal DVT and can progress to proximal DVT or even PE [21]. Other studies have reported that CMVT is found in 23–41% of patients suspected of having DVT and in 47–79% of patients with confirmed DVT [22–24]. However, the reports on CMVT of patients admitted to ICU with free flaps after head and neck surgery are very limited.

The primary aim of the present study was to investigate the risk factors for postoperative complications related to free flap reconstruction for patients admitted to the ICU. The second aim was to investigate the factors associated with thrombotic complications after free flap surgery in head and neck cancer patients.

## Methods

**Fig. 1:**
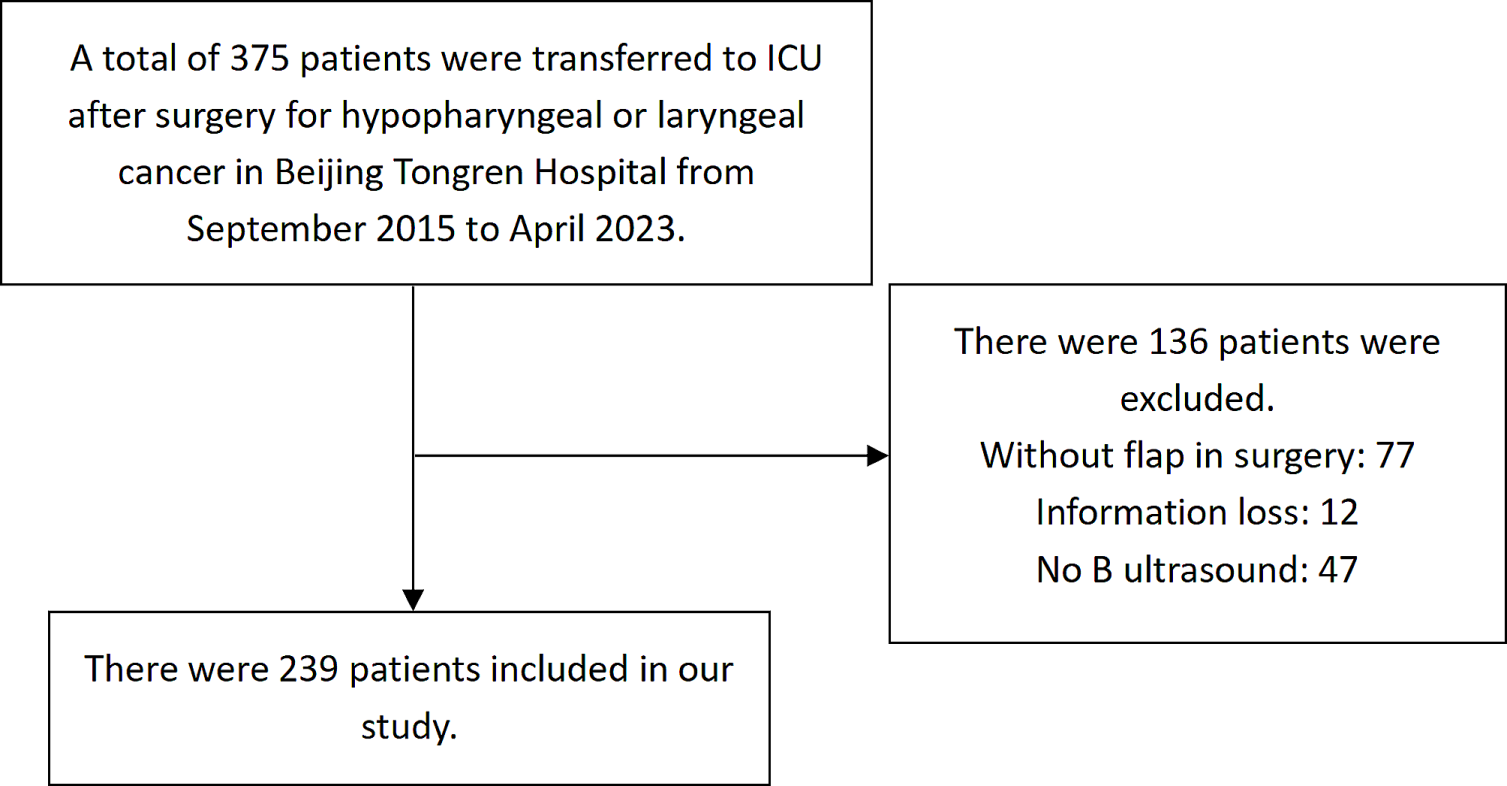
Flow chart of patient selection and reasons for exclusion. In the study, a total of 375 patients were initially included. However, 77 patients did not undergo flap surgery, 12 patients had incomplete information available, and 47 patients were lacking ultrasound results. After accounting for these exclusions, a final sample of 239 patients was included in the study. ICU: Intensive Care Unit

From September 2015 to April 2023, we collected all head and neck cancer patients with free flap reconstruction admitted to the ICU after surgery at Beijing Tongren Hospital, Capital Medical University. This study was approved by the ethical committee of Beijing Tongren Hospital. The informed consent was waived by the ethical committee of Beijing Tongren Hospital due to the retrospective design. Patients were transferred to ICU after surgery when met one or more of the following criteria: (1) Combined organ reserve insufficiency, such as cardiopulmonary diseases with monitoring before surgery, renal insufficiency; (2) Age > 70 years; (3) Intraoperative bleeding > 1000 ml or duration of surgery > 6 h; (4) Intraoperative complications such as circulatory instability or hypoxia. (Fig. 1)

After the operation, the airway condition was evaluated, and the patients were weaned off the ventilator after a spontaneous breathing test. After tracheal intubation was removed from the intubation, the airway was continuously oxygenated and humidified to maintain the airway channel. The treatment in the ICU included maintaining effective circulating blood volume, internal environment stability, self-control pump intravenous analgesia, and prevention and treatment of infection. The patient was transferred to the general ward for continued treatment after stable general condition.

Patient information was collected from all patients with head and neck tumors who underwent free flaps and were transferred to the ICU after surgery at Beijing Tongren Hospital. The variables were extracted from electrical medical records (EMR). The Epidata software was used to extract the content. Two investigators independently extracted demographic and clinical data including medical history, anesthesia notes, surgical records, imaging reports, laboratory results, hospital progress notes, and discharge summaries. Discrepancies were initially cross-checked against the original data, and if they could not be reconciled, a third expert was needed to provide a final judgment. The preoperative variables included surgical age, body mass index (BMI), sex, smoking history, drinking history, coexisting diseases (hypertension, diabetes, kidney disease, heart disease), radiotherapy, and chemotherapy. The intraoperative variables included operation duration, vasopressor, blood loss, fluid input (ml/kg.h), fluid balance (ml/kg.h), and femoral vein catheterization. Postoperative variables included vasopressor, anticoagulation, dextran, fluid input (ml/kg.h), and fluid balance (ml/kg.h) during the first day in ICU. The mental state, blood pressure, heart rate, respiration, blood oxygen saturation, central venous pressure, 24-hour intake and output, blood gas analysis, blood routine, blood electrolyte, peripheral blood glucose, and liver and kidney function were monitored during the first 24 h in ICU.

The main outcome was postoperative complications related to free flap reconstruction, including flap necrosis, bleeding, fistula, and infection based on medical records. The skin flap color, blood supply, and wound negative pressure drainage were observed every four hours. The secondary outcome was CMVT based on a B-ultrasound of the lower extremity. CMVT showed no echo or low echo in the lumen and the lumen could not be compressed. Bedside ultrasound examination of the lower extremity deep vein was performed on the day or the first day after the operation by ultrasound physicians.

The normality of variable distribution was evaluated using the Kolmogorov-Smimov test. The variables with normal distribution were described using means ± standard deviations, and the variables with non-normal distribution were described using median (interquartile range, IQR). To provide an optimal threshold for the use of the diagnostic test for classification, the continuous variables were stratified and the cutoff values were determined from the Yoden index in the receiver operating characteristic analysis. The chi-square or Fisher exact test was used to compare the single variable between groups. The variables with *P* < 0.05 in the univariate analysis were put into the multivariate logistic regression model. All statistics were performed in the SPSS 22.0 version (IBM, Amenk, NY, USA), and false discovery rate correction for multiple comparisons was applied with a significance level of P value < 0.05.

## Results

### General population

Of the 375 patients who were transferred to the ICU after surgery on head and neck cancers with free flaps reconstruction, 239 patients were finally included in the study after exclusion (Fig. 1). The percentage of male patients was 83.7% (*n* = 200), and female patients was 16.3% (*n* = 39). The median (IQR) age was 55.0 (50.0–63.0) years. The mean (± standard deviation) BMI was 23.1 ± 3.2 kg/m^2^. Ninety-two (38.5%) patients had a history of alcohol intake and 112 (46.9%) patients had a history of smoking. Among the included patients, a total of 58 (24.3%) patients had hypertension; 17 (7.1%) patients had diabetes; 10 (4.2%) patients had coronary heart disease. Besides, 42 (17.6%) patients had taken radiation therapy, and 72 (30.1%) patients had taken chemotherapy. Among the flap-related complications observed in all patients, there were 8 cases (3.34%) of flap necrosis, 18 cases (7.53%) of pharyngeal fistula, 22 cases (9.21%) of incision infection, and 6 cases (2.51%) of hemorrhage. Additional preoperative information is provided in Table 1.

**Table 1:** General information of the patients

General Information	*N* = 239
Surgical age, yr	55.0 (50.0–63.0)
BMI, kg/m^2^	23.1 ± 3.2
Male	200 (83.7%)
Drinking	92 (38.5%)
Smoking	112 (46.9%)
Radiotherapy	42 (17.6%)
Chemotherapy	72 (30.1%)
Hypertension	58 (24.3%)
Diabetes	17 (7.1%)
Coronary heart disease	10 (4.2%)
Operation duration, hours	8.3 ± 2.9
Vasopressor in surgery	24 (10.0%)
Blood loss during surgery, ml	300 (150–750)
Fluid input during surgery, ml	5.4 (4.2-7.0)
Fluid balance during surgery, ml	5.3 ± 2.5
Femoral vein catheterization	96 (40.2%)
Vasopressor in ICU	34 (14.2%)
Anticoagulation	24 (10.1%)
Dextran	49 (20.5%)
Fluid input during the first day in ICU, ml/kg.h	3.3 (2.7–3.9)
Fluid balance during the first day in ICU, ml/kg.h	1.1 ± 1.1
Postoperative day-1 WBC, ×10^9^/L	10.8 (9.0-13.4) ×10^9^
Postoperative day-1 NEU%	86.6% (82.7–90.0%)
Postoperative day-1 CRP, mg/L	27.4 (17.8–43.1)
Postoperative day-1 Albumin, g/L	29.7 ± 3.8
Postoperative day-1 Hemoglobin, g/L	106.4 ± 16.9

Data are presented by mean ± standard deviation, median (interquartile range), or No. (%)

BMI: Body mass index, ICU: Intensive Care Unit, WBC: White blood cell, NEU%: neutrophilic granulocyte percentage, CRP: C-reactive protein

**Table 2:** Comparison of the patient characteristics between those with and without flap-related complications

Variables	No complications (*N* = 201)	Complications (*N* = 38)	*P* value
Surgical age (> 60 y)	66 (32.0%)	12 (31.6%)	0.864
BMI (> 18 kg/m^2^)	191 (95%)	28 (73.7%)	**0.000** ^**^
Male	169 (84.1%)	31 (81.6%)	0.702
Drinking	76 (37.8%)	16 (42.1%)	0.618
Smoking	89 (44.3%)	23 (60.5%)	0.066
Radiotherapy	31 (15.4%)	11 (28.9%)	**0.045** ^*^
Chemotherapy	59 (29.4%)	13 (34.2%)	0.550
Hypertension	47 (23.4%)	11 (28.9%)	0.463
Diabetes	17 (7.9%)	0 (0.0%)	0.312
Chronic kidney disease	4 (1.9%)	0 (0.0%)	1.000
Coronary heart disease	9 (4.2%)	1 (4.2%)	1.000
Operation duration (> 9 h)	48 (23.9%)	16 (42.1%)	**0.020** ^*^
Vasopressor in surgery	18 (9.0%)	6 (15.8%)	0.322
Blood loss during surgery (> 650 ml)	51 (25.4%)	3 (7.9%)	**0.018** ^*^
Fluid input during surgery (> 3.53 ml/kg.h)	182 (91.0%)	29 (76.3%)	**0.019** ^*^
Fluid balance during surgery (> 6.85 ml/kg.h)	49 (24.4%)	4 (10.5%)	0.059
Femoral vein catheterization	81 (40.3%)	15 (39.5%)	0.924
Vasopressor in ICU	26 (12.9%)	8 (21.1%)	0.189
Anticoagulation	17 (8.5%)	7 (18.4%)	0.117
Dextran	44 (21.9%)	5 (13.2%)	0.221
Fluid input during the first day in ICU (> 2.41 ml/kg.h)	170 (84.6%)	25 (65.8%)	**0.006** ^*^
Fluid balance during the first day in ICU (> 0.26 ml/kg.h)	169 (84.1%)	28 (73.7%)	0.123
Postoperative day-1 WBC (> 15.39 × 10^9^/L)	25 (12.5%)	9 (23.7%)	0.071
Postoperative day-1 NEU% (> 88.55%)	78 (38.8%)	9 (23.7%)	0.076
Postoperative day-1 CRP (> 33.38 mg/L)	70 (36.5%)	26 (68.4%)	**0.000** ^**^
Postoperative day-1 Albumin (> 32.05 g/L)	54 (27.6%)	6 (15.8%)	0.129
Postoperative day-1 Hemoglobin (> 102.5 g/L)	116 (57.7%)	15 (39.5%)	**0.038** ^*^

BMI: Body mass index, ICU: Intensive Care Unit, WBC: White blood cell, NEU%: neutrophilic granulocyte percentage, CRP: C-reactive protein

^*^: *P* < 0.05

^**^: *P* < 0.001

### Intraoperative variables

There were no deaths recorded in the medical chart. The average operative time was 8.3 ± 2.9 h. There was a surgical blood loss of 300 (150–750) milliliters. During surgery, the fluid input was 5.4 (4.2-7.0) ml/kg.h, and the average fluid balance was 5.3 ± 2.5 ml/kg.h. Furthermore, femoral venous catheterization was performed in 96 (40.2%) patients. Other intraoperative information is presented in Table 1.

### ICU variables

The median length of ICU stay was 1 day (IQR, 1–2 days). Thirty-four (14.2%) patients had low blood pressure postoperatively and were treated with vasopressor in ICU. In addition, 24 (10.1%) patients and 49 (20.5%) patients received anticoagulation and dextran respectively. The mean fluid balance during ICU was 1.1 ± 1.1 ml/kg.h, and the median (IQR) fluid input was 3.3 (2.7–3.9) ml/kg.h during the first day in ICU. The laboratory indicators on the first postoperative day were as follows: white blood cell (WBC) was 10.8 (9.0-13.4) ×10^9^/L, neutrophilic granulocyte percentage (NEU%) was 86.6% (82.7–90.0%), and C-reactive protein (CRP) was 27.4 (17.8–43.1) mg/L; the mean albumin was 29.7 ± 3.8 g/L, and the average hemoglobin was 106.4 ± 16.9 g/L. Further ICU information is provided in Table 1.

### Free flap-related complications and influencing factors

**Fig. 2:**
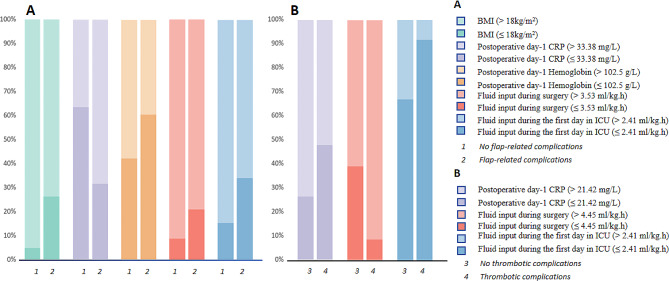
Differences in risk factors for different complications. **(A)** The multivariate analysis showed BMI, postoperative day-1 CRP, postoperative day-1 hemoglobin, fluid input during surgery, and fluid input during the first day in ICU were independently associated with postoperative complications related to free flap reconstruction. **(B)** The multivariate analysis suggested that fluid input during surgery, fluid input during the first day in ICU, and postoperative day-1 CRP were independently associated with thrombotic complications. BMI: Body mass index, ICU: Intensive Care Unit, CRP: C-reactive protein

Prognostic factors for the postoperative complications related to free flap reconstruction were further analyzed. Initially, continuous variables were converted into categorical variables. The univariate analysis was performed and the results suggested the following variables were significant: surgical age (> 60 y), BMI (> 18 kg/m^2^), radiotherapy, operation duration (> 9.8 h), blood loss during surgery (> 650 ml), colloid input during surgery (> 2.3 ml/kg.h), crystalloid input during ICU in 24 h (> 2.4 ml/kg.h), postoperative CRP (> 33.4 mg/L), postoperative HGB (> 102.5 g/L, *P* < 0.05, Table 2). The multivariate analysis showed BMI (OR = 0.183, 95% CI = 3.948–44.249, *P* < 0.001, Table 3), postoperative day-1 CRP (OR = 0.291, 95% CI = 0.123–0.688, *P* = 0.005, Table 3), postoperative day-1 hemoglobin (OR = 3.265, 95% CI = 1.297–8.219, *P* = 0.012, Table 3), fluid input during surgery (OR = 0.183, 95% CI = 0.059–0.573, *P* = 0.004, Table 3), and fluid input during the first day in ICU (OR = 3.718, 95% CI = 1.374–10.061, *P* = 0.010, Table 3) were independently associated with postoperative complications related to free flap reconstruction. (Fig. 2A)

**Table 3:** Predictors of flap-related complications on multivariate analysis

Variables	OR	95%CI	*P* value
Radiotherapy	0.403	0.151–1.080	0.071
BMI (> 18 kg/m^2^)	0.183	3.948–44.249	**0.000** ^**^
Operation duration (> 9 h)	0.476	0.196–1.154	0.476
Blood loss during surgery (> 650 ml)	3.936	0.951–16.285	0.059
Postoperative day-1 CRP (> 33.38 mg/L)	0.291	0.123–0.688	**0.005** ^*^
Postoperative day-1 hemoglobin (> 102.5 g/L)	3.265	1.297–8.219	**0.012** ^*^
Fluid input during surgery (> 3.53 ml/kg.h)	0.183	0.059–0.573	**0.004** ^*^
Fluid input during the first day in ICU (> 2.41 ml/kg.h)	3.718	1.374–10.061	**0.010** ^*^

BMI: Body mass index, CRP: C-reactive protein, ICU: Intensive Care Unit

^*^: *P* < 0.05

^**^: *P* < 0.001

### Thrombotic complications and influencing factors

Prognostic factors for CMVT were also analyzed. Continuous variables were transformed into categorical variables. Through univariate analysis, the following factors showed a significant difference between the patients with or without thrombotic complications: fluid input during surgery (> 4.5 ml/kg.h), fluid input during the first day in ICU (> 3.8 ml/kg.h), and postoperative day-1 CRP (> 21.4 mg/L, *P* < 0.05, Table 4). The multivariate analysis suggested that fluid input during surgery (OR = 0.374, 95% CI = 0.150–0.929, *P* = 0.034, Table 5), fluid input during the first day in ICU (OR = 0.215, 95% CI = 0.048–0.965, *P* = 0.045, Table 5), and postoperative day-1 CRP (OR = 6.769, 95% CI = 1.521–30.117, *P* = 0.012, Table 5) were independently associated with thrombotic complications. (Fig. 2B)

**Table 4:** Comparison of the patient characteristics between those with and without calf muscle vein thrombosis

Variables	No complications (*N* = 215)	Complications (*N* = 24)	*P* value
Surgical age (> 60 y)	66 (84.6%)	12 (15.4%)	0.056
BMI (> 18 kg/m^2^)	202 (89.4%)	24 (10.6%)	0.445
Male	178 (82.8%)	22 (91.7%)	0.409
Drinking	83 (38.6%))	9 (37.5%)	0.916
Smoking	98 (45.6%)	14 (58.3%)	0.235
Radiotherapy	40 (90.91%)	9 (75.00%)	0.140
Chemotherapy	65 (30.5%)	7 (29.2%)	0.914
Hypertension	53 (24.7%)	5 (20.8%)	0.679
Diabetes	17 (7.9%)	0 (0.0%)	0.312
Chronic kidney disease	4 (1.9%)	0 (0.0%)	1.000
Coronary heart disease	9 (4.2%)	1 (4.2%)	1.000
Operation duration (> 9 h)	55 (25.6%)	9 (37.5%)	0.211
Vasopressor in surgery	21 (9.8%)	3 (12.5%)	0.949
Blood loss during surgery (> 75 ml)	51 (23.7%)	3 (12.5%)	0.212
Fluid input during surgery (> 4.45 ml/kg.h)	156 (73.2%)	12 (50%)	**0.018** ^*^
Fluid balance during surgery (> 4.85 ml/kg.h)	48 (22.3%)	5 (20.8%)	0.867
Femoral vein catheterization	83 (38.6%)	13 (54.2%)	0.140
Vasopressor in ICU	28 (13.0%)	6 (25.0%)	0.199
Anticoagulation	21 (9.8%)	3 (13.0%)	0.895
Dextran	48 (22.3%)	1 (4.2%)	0.068
Fluid input during the first day in ICU (> 3.76 ml/kg.h)	71 (33.0%)	2 (8.3%)	**0.013** ^*^
Fluid balance during the first day in ICU (> 1.91 ml/kg.h)	177 (82.3%)	20 (83.3%)	1.000
Postoperative day-1 WBC (> 14.81 × 10^9^/L)	33 (15.4%)	1 (4.2%)	0.235
Postoperative day-1 NEU% (> 88.55%)	82 (38.1%)	5 (20.8%)	0.095
Postoperative day-1 CRP (> 21.42 mg/L)	82 (39.6%)	14 (60.9%)	**0.050** ^*^
Postoperative day-1 Albumin (> 30.45 g/L)	57 (27.1%)	3 (12.5%)	0.120
Postoperative day-1 Hemoglobin (> 94.5 g/L)	116 (54.0%)	15 (62.5%)	0.425

BMI: Body mass index, ICU: Intensive Care Unit, WBC: White blood cell, NEU%: neutrophilic granulocyte percentage, CRP: C-reactive protein

^*^: *P* < 0.05

**Table 5:** Predictors of thrombotic complications on multivariate analysis

Variables	OR	95%CI	*P* value
Fluid input during surgery (> 4.45 ml/kg.h)	0.374	0.150–0.929	**0.034** ^*^
Fluid input during the first day in ICU (> 3.76 ml/kg.h)	0.215	0.048–0.965	**0.045** ^*^
Postoperative day-1 CRP (> 21.42 mg/L)	6.769	1.521–30.117	**0.012** ^*^

ICU: Intensive Care Unit, Postop 1: postoperative day 1, CRP: C-reactive protein

CRP: C-Reactive Protein

^*^: *P* < 0.05

## Discussion

The present study showed that fluid input during operation and the first day in ICU were independently associated with postoperative complications related to free flap reconstruction and CMVT, which suggested that vigilant fluid management would have a beneficial effect for patients with head and neck cancer who underwent resection with free flap reconstruction. Besides, lower BMI was at a higher risk of postoperative complications, whereas those with higher BMI, postoperative day-1 hemoglobin, and lower postoperative day-1 CRP had a lower risk for postoperative complications. Low postoperative day-1 CRP was associated with low thrombotic complications.

Perioperative variables, including surgical age, sex, smoking, drinking, chemotherapy, and basic diseases, were not associated with postoperative complications with multivariate analysis. These results are consistent with the findings of previous studies [6, 25]. Besides, some studies have shown that radiotherapy can significantly increase the rate of free flap complications [26, 27], while others have shown no significant effect [28, 29]. Our results showed that radiotherapy was related to the higher incidence of free flap complications but was not statistically significant. Although patients with a high BMI are generally at an increased risk for postoperative complications [30], the low BMI patients showed more complications related to free flap reconstruction in this study. Previous studies showed the obesity paradox widely existed in ICU patients [31]. Underweight patients have a higher chance of a large number of comorbidities, including poor lifestyle habits such as smoking and alcohol consumption; general health indicators such as anemia and impaired functional status; and measures of disease severity, including disseminated cancer and prior chemotherapy [32]. Heo et al. also suggested that patients with low BMI combined more complications after free flap surgery [33].

In the perioperative period, vasopressors were necessary to ensure perfusion. This study showed that the intraoperative and postoperative vasopressors were unrelated to the increasing risk of free flap complications, consistent with previously reported results [34, 35]. The dose of norepinephrine applied to our included patients ranged from 0 to 0.2ug/kg. min, thus indicating that low-dose vasoconstrictors did not increase the rate of free flap-related complications. The significance of fluid management in the perioperative management of patients undergoing free flap surgery is still under debate. The High-volume hemodilution obtained by fluid infusions increased blood flow to normal tissues and could even increase blood flow to the free flap [36]. Inadequate infusion would result in insufficient effective perfusion of the free flap [37]. In the present study, the fluid infusion rate during surgery and the first day in the ICU was an identified risk factor that predicted postoperative complications related to free flap reconstruction. Our results showed that fluid input > 3.53 ml/kg.h during surgery and > 2.41 ml/kg.h during the first day in ICU could reduce free flap-related complications. Zhong et al. [12]. recommend that the rate of crystalloid replenishment should be controlled at 3.5 to 6 mL/kg per hour during the 24-hour perioperative period. Clark et al. [7]. found that crystal fluid intake over 130 ml/kg/24 hours was an independent predictor of postoperative complications. The results suggested that an appropriate increase in fluid intake, not only intraoperatively but also postoperatively, can reduce complications in head and neck patients undergoing free tissue transfer, and the conclusion was consistent with C. Wang et al. [8]. Therefore, it is recommended to increase fluid intake appropriately in the postoperative management of patients undergoing free flap reconstruction for head and neck cancer to help reduce the risk of complications.

The fluid input during surgery and the first day in ICU was also associated with thrombosis. It is well established that thrombus formation requires three factors: venous blood flow stasis, vascular endothelial injury, and blood hypercoagulability [38]. The effect of crystalloids on coagulation was thought to be primarily dilutional and appeared proportionate to the degree of dilution [39]. Synthetic colloid-containing fluids potentially contribute to coagulopathy through both dilutional and non-dilutional mechanisms, including direct effects on platelets and coagulation factors [39]. 5% albumin has also been shown to reduce coagulation [40, 41]. Therefore, appropriate postoperative rehydration to avoid hemoconcentration can reduce the risk of blood hypercoagulation secondary to thrombosis. Furthermore, once the free flap fails, the hypercoagulable state is one of the strongest factors associated with low salvage rates [42].

The body produces CRP in response to infection, inflammation, malignancy, and trauma, making it a widely recognized clinical marker for detecting infection [43]. The CRP could increase dramatically within 24–72 h of severe tissue damage such as trauma and progressive cancer [44]. Thus, studies have been conducted to examine the association between CRP levels and complications following free flap surgery. A study that examined 25 postoperative patients with head and neck free flaps and continuously monitored daily postoperative CRP levels found that trends in postoperative CRP were predictive of complications [45]. They found that CRP levels need to be monitored for at least 4 days after surgery and a second elevated CRP indicated a high likelihood of flap complication. However, Koerdt et al. [46]. didn’t find statistically significant differences in serum levels between patients with postoperative reduced flap perfusion and patients without low perfusion. In this study, we collected serum CRP levels on the first postoperative day, indicated as an independent risk factor for predicting free flap related complications. Significantly elevated CRP in the early stages may indicate severe trauma or acute infection, and this leads to a high complication rate. Besides, a significant decrease in postoperative day-1 hemoglobin was a potential predictor of free flap complications, which was consistent with Wang’s study [6]. Hemoglobin would act as a surrogate for nutritional and general health status since it correlated significantly with low weight and percentage weight loss (*P* < 0.05) [7]. Kim et al. [47]. also indicated perioperative lowest hemoglobin was a significant predictor of flap failure. Hill et al. [48]. reported a similar observation in their study and put forward a potential physiological mechanism suggesting that in cases of anemia, the decrease in blood viscosity can change laminar flow to turbulent flow, increasing the risk of thrombosis, which could result in partial or complete flap failure. In patients who have undergone free flap surgery, monitoring and closely evaluating CRP and hemoglobin levels are crucial as they serve as key indicators of postoperative recovery and possible complications.

Our study suggested elevated postoperative day-1 CRP levels indicated an increased risk of postoperative thrombosis. The link between DVT and inflammation facilitating thrombosis was discussed in several studies [49–51]. Higher serum levels of inflammatory cytokines, including CRP, were associated with a higher risk of DVT, according to Ma et al. [50]. Inflammatory biomarkers including CRP were found to be associated with VTE, but it was inconclusive that elevations in these biomarkers were from systemic inflammation or played an integral part in the formation of thrombosis [52]. Regardless of the mechanism, CRP levels were elevated in the early stages of thrombosis.

### Limitations

While our study was a meaningful step toward postoperative ICU management of patients with combined free flap transplantation for head and neck cancer, there were limitations to this strategy. First, this study was limited by its retrospective design, which cannot control for missing data, bias, and variation in practice or protocol, and these factors may affect the accuracy of the results. Second, complications had a lower probability of occurring, resulting in a larger difference in sample size between groups. Third, the patients in this study were operated on by different surgeons, and differences in surgeon competence could also contribute to differences in postoperative complications. Finally, the sample of the patients was from a single institution, thus the scope of the study is limited, with difficulty in covering different regions and populations. In this study, two kinds of high-incidence complications after flap surgery were analyzed separately to explore the differences in risk factors of different complications. We refrained from performing subgroup analyses on age, gender, and other variables due to the limited sample sizes within these subgroups or significant disparities in their distribution, which could compromise the robustness of our findings. The results of risk factor differences between groups with or without surgical complications were presented in bar charts for easy understanding. Other potential risk factors could be explored in future studies. Additionally, more research could be done to investigate the mechanisms underlying the association between hemoglobin and CRP levels and postoperative complications. Future prospective studies of a wider and more diverse research population can be beneficial in confirming these findings.

## Conclusion

The postoperative complications related to free flap reconstruction were common for head and neck cancer patients admitted to the ICU. The low BMI, low postoperative day-1 hemoglobin, high CRP, and inadequate fluid input during operation and the first day in ICU were associated with poor outcomes related to free flap reconstruction.

## Data Availability

Data can be requested from the corresponding author if necessary.
